# The Molecular Exposome of Visible Age Reversal: From Organ–Skin Axes to Regenerative Aesthetics

**DOI:** 10.3390/molecules31071147

**Published:** 2026-03-31

**Authors:** Hidekazu Yamada

**Affiliations:** Anti-Aging Center, Faculty of Medicine 3-1, Kindai University, Chayayama-dai, Minami-ku, Sakai 590-0115, Osaka, Japan; yamadahi@med.kindai.ac.jp; Tel.: +81-722887222

**Keywords:** organ–skin axis, epigenetic aging, health capital, klotho (KL1), myokines, NAD+, GLP-1, mechanotransduction, PLLA, exosomes, regenerative aesthetics

## Abstract

Cosmetic dermatology has largely focused on topical applications targeting the stratum corneum. However, emerging evidence suggests that visible aging is a systemic readout of internal “organ clocks” and molecular dysregulation across the epidermis and dermis. This review proposes an “inside–out strategy” that seeks to re-conceptualize aesthetic vitality as a measurable indicator of systemic physiological resilience. The author describes theoretically proposed organ–skin axes, including the role of molecular signaling of kidney-derived klotho (KL1 fragment) via FGFR1-α–klotho complexes and muscle-derived irisin through the AMPK/PGC-1-α pathway in modulating skin homeostasis. Drawing on recent breakthroughs in non-human primate models (2023–2025), this synthesis explores the potential of systemic interventions—including nicotinamide adenine dinucleotide (NAD+) precursors (sirtuin 1 SIRT1 activators), senolytics (targeting BCL-2/p16), and glucagon-like peptide-1 (GLP-1) receptor agonists—as candidates to potentially synchronize these internal clocks. Furthermore, the review identifies direct regenerative interventions, such as retinoids (RAR/RXR signaling), chemical peels (HIF-1-α induction), exosomes (miR-21/29 delivery), and poly-L-lactic acid PLLA (mechanotransduction via YAP/TAZ), positioning them as potential physical and chemical epigenetic modulators that may support the restoration of cellular transcriptional fidelity. This article proposes a new paradigm for regenerative aesthetics that focuses on restoring the youthful phenotype by optimizing systemic molecular crosstalk and epigenetic transcriptional fidelity.

## 1. Introduction: The Limits of Topical Approaches

According to the traditional “wear and tear” theory of aging, biological systems inevitably degrade, much like mechanical parts [1]. However, the 21st-century “information theory of aging” has proposed a re-conceptualization of aging as a loss of epigenetic transcriptional fidelity—a corruption of the cellular software rather than irreversible hardware damage [2]. Central to this theory is the concept of mesenchymal drift and loss of cellular identity, hypothesized to be driven by the relocation of chromatin-modifying enzymes, such as sirtuins, from their original loci to sites of DNA damage [2,3].

**Figure 1:**
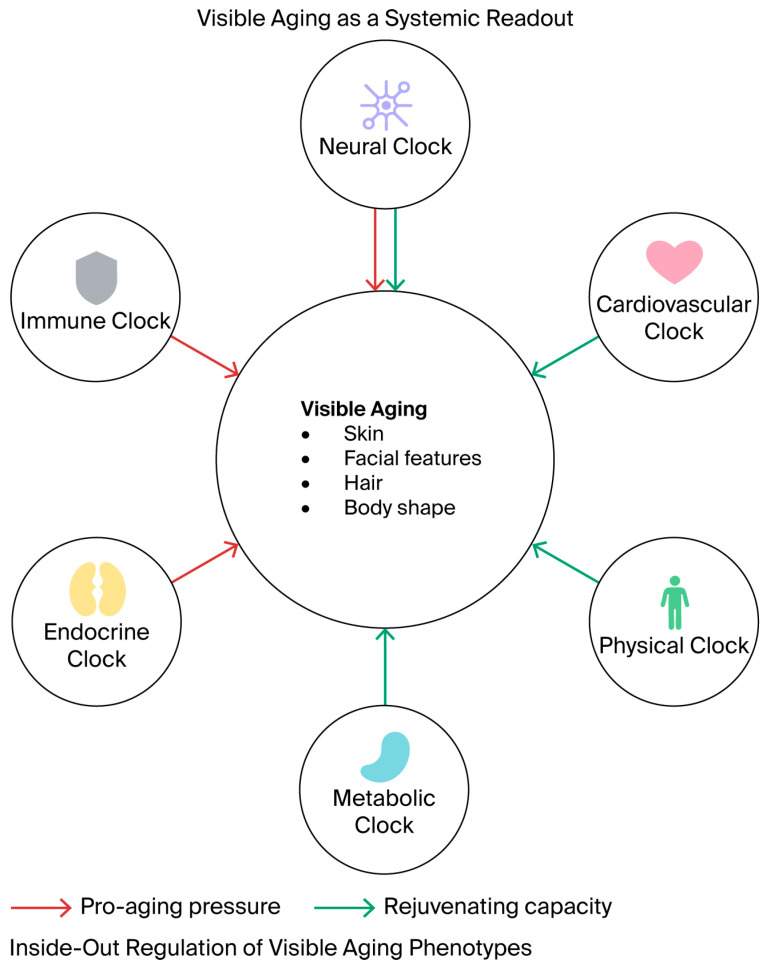
The organ–skin molecular crosstalk. Visible aging is conceptualized not merely as a chronological marker but serves as a phenotypic readout of systemic biological health (akin to an Epigenetic clock). The skin functions as a “biological interface “ that integrates convergent molecular signals from four primary organ systems: the brain axis (regulating stress via cortisol and social connection via oxytocin), the gut axis (modulating inflammation via microbiome metabolites like SCFAs and LPS), the muscle axis (providing restorative signals via myokines, such as irisin), and the kidney axis (defending against oxidative stress via klotho and vitamin D). These systemic inputs collectively shape four key components of appearance: skin quality, facial morphology, hair health, and body shape. Red arrows indicate pro-aging pressures and negative systemic influences, while green arrows represent rejuvenating capacity and protective molecular signaling.

This paradigm shift is critical to dermatology. Conventional cosmetic approaches have predominantly focused on “camouflage” or topical moisture replenishment. However, visible aging—characterized by wrinkles, sagging, and pathological pigmentation—is increasingly recognized not as a localized event, but as the macroscopic manifestation of systemic inflammaging and the desynchronization of internal “organ clocks” [4,5]. Recent longitudinal studies have demonstrated that individuals who look older than their chronological age exhibit accelerated biological aging across multiple organ systems, quantifiable using epigenetic clocks such as DunedinPACE [5,6]. This review theoretically proposed the molecular crosstalk between internal organs and the skin (the organ–skin axes). Specifically, this review elucidates how systemic factors like klotho [7,8,9], irisin [10], and NAD+ metabolites [2,3] may chemically modulate skin homeostasis. Furthermore, this article proposes redefining direct regenerative interventions—including retinoids, exosomes, and PLLA [11]—as epigenetic modulators that might support the restoration of transcriptional fidelity via RAR/RXR and YAP/TAZ [12,13] signaling (Figure 1).

## 2. Methodology

A comprehensive literature search was conducted using databases including PubMed, Google Scholar, and Scopus, spanning publications from 2015 to 2025. Primary search terms included “epigenetic aging,” “organ–skin axis,” “mechanotransduction,” and “regenerative aesthetics”. Selection criteria focused on recent breakthroughs in non-human primate models (2023–2025), high-impact clinical trials, and molecular biology studies investigating systemic clock synchronization and cellular transcriptional fidelity.

## 3. The Organ–Skin Axis: A Systemic Framework

### 3.1. The Brain–Gut–Skin Trinary Loop: Integrated Signaling Pathways

**Figure 2:**
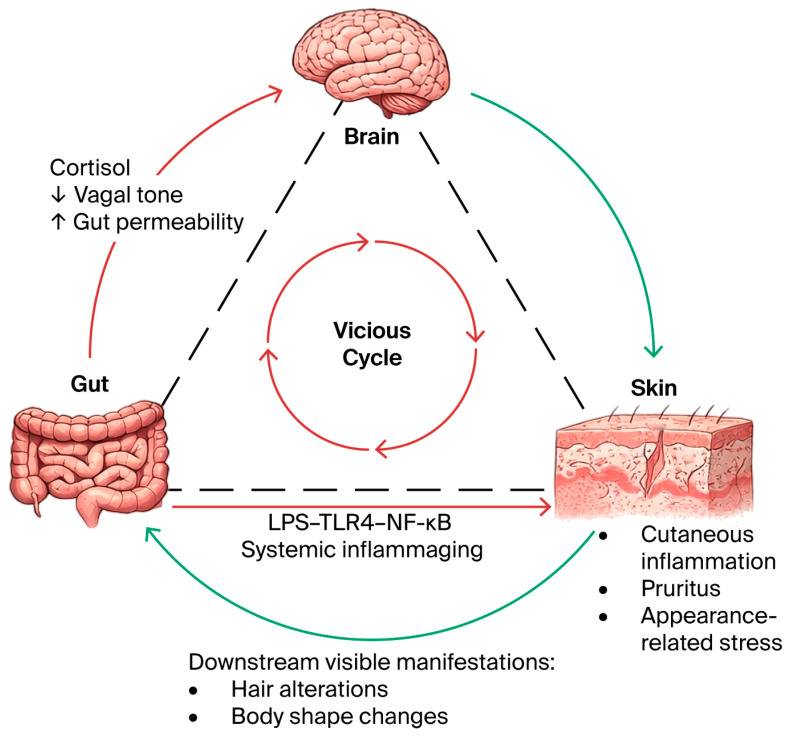
The brain–gut–skin trinary loop. Psychological stress and gut dysbiosis are hypothesized to form a self-reinforcing inflammatory loop involving the brain, gut, and skin. At the molecular level, intestinal hyperpermeability may facilitate the translocation of lipopolysaccharides (LPS), which bind to TLR4 and activate the NF-kappaB pathway, potentially driving systemic inflammaging. This dysregulated circuit has been associated with the acceleration of cutaneous aging phenotypes (xerosis, pruritus) and may contribute to systemic manifestations, including potential hair alterations and body shape changes. “Dashed lines represent proposed bidirectional feedback loops and potential molecular interactions between the gut, brain, and skin.”

The skin, brain, and gut share a common embryological origin, forming a synchronized trinary loop that is hypothesized to maintain homeostasis through complex molecular signaling [5]. In the proposed skin–brain axis, sensory inputs such as affective touch engage low-threshold mechanoreceptors, which project to the insular cortex and may trigger the systemic release of oxytocin and dopamine. This neuro-endocrine response has been observed to downregulate the hypothalamic–pituitary–adrenal (HPA) axis, reducing systemic cortisol and suppressing pro-inflammatory cytokines like IL-6 and TNF-alpha in clinical and experimental settings [14]. Similarly, cutaneous thermosensors transmit environmental cues to the hypothalamus to modulate the circadian clock and sleep architecture, which are critical for nocturnal DNA repair (Figure 2) [15].

Conversely, the brain–skin axis operates through neuro-endocrine effectors. Clinical evidence suggests that chronic psychological stress elevates systemic cortisol, which binds to glucocorticoid receptors in keratinocytes to inhibit the transcription of essential barrier components such as hyaluronan synthase 2 (HAS2) and filaggrin [16]. This neurogenic signaling is thought to be further exacerbated by the release of Substance P and CGRP from peripheral nerve endings, triggering mast cell degranulation. These signals also influence the gut, where sympathetic overactivity increases intestinal permeability. This “leaky gut” state enables the translocation of luminal endotoxins into circulation, such as lipopolysaccharides (LPS). These endotoxins subsequently bind to TLR4 on dermal fibroblasts, activating the MyD88-dependent NF-kappaB pathway and potentially accelerating systemic inflammaging. This trinary cycle is completed by gut-derived metabolites, particularly short-chain fatty acids (SCFAs), which have been shown in animal models to cross the blood–brain barrier to support neuroplasticity, thus potentially reinforcing the skin’s structural resilience from within [17].

### 3.2. Clinical Manifestations of the Trinary Loop in Aesthetics

Disruption of the abovementioned molecular pathways manifests as distinct clinical phenotypes. Stress-induced xerosis is hypothesized to be driven by cortisol-mediated suppression of lipid and hyaluronic acid synthesis, presenting as dullness resistant to topical moisturizers. Furthermore, the “hormone clock” is thought to significantly tunes this axis, as the periodic decline in estrogen during the menstrual phase has been associated with a transient barrier drift and increased susceptibility to the exposome [10].

### 3.3. Endocrine Support: The Muscle and Kidney Axes

Beyond the trinary loop, the skin appears to rely on endocrine support from the musculoskeletal and renal systems. Skeletal muscle functions as a secretory organ, releasing myokines such as irisin during contraction. Experimental models suggest that irisin enters the systemic circulation and binds to integrin α receptors on dermal fibroblasts, where it may enhance mitochondrial biogenesis via PGC-1-α signaling and mitigates oxidative stress [14]. Consequently, age-related sarcopenia could lead to systemic deficiency in these “youth signals,” potentially contributing to dermal sagging.

Parallel to the muscle–skin axis, the renal system provides essential longevity factors. The kidney-derived master anti-aging protein klotho is proposed to serve as a systemic “shield” for the skin [7]. Recent landmark studies (2023–2025) in aged non-human primates have demonstrated that systemic klotho levels are linked to cognitive resilience [13], providing a basis for its potential role in systemic rejuvenation. This proposed dermo-protective effect is mediated by the circulating KL1 fragment, which is hypothesized to act as a humoral messenger that preserves the integrity of the extracellular matrix (ECM) by modulating the TGF-β and Wnt signaling pathways. A decline in renal function—the “kidney clock”—may deplete this klotho shield, potentially accelerating dermal atrophy [9,18]. Kidney-derived KL1 signaling provides a molecular link for our inside–out model, suggesting that visible skin health may serve as a readout of the systemic abundance of circulating longevity factors.

## 4. Environmental and Social Modulation of Epigenetic Aging

### 4.1. Social Genomics and Epigenetic Plasticity

Emerging research in social genomics establishes the social environment as a critical biological determinant of the aging rate. Recent studies using the DunedinPACE clock have shown that environmental stressors can lead to a measurable acceleration in the pace of biological aging [6,19]. Notably, it has been established that biological age is not a static trajectory but can be transiently increased by systemic stress and subsequently restored upon recovery, highlighting the remarkable plasticity of the epigenetic landscape [20].

The author suggests that positive aesthetic perception—mediated by the “Paris Effect”—engages the medial orbitofrontal cortex (mOFC). While the neurobiology of beauty has been linked to mOFC activation [21], this synthesis further proposes that such activation may serve as a neuro-biological stabilizer to modulate HPA axis reactivity, potentially mitigating the deleterious effects of environmental stress on skin homeostasis.

### 4.2. Mechanobiological Modulation of the Skin’s Epigenetic Landscape

The transduction of physical signals from facial expressions is increasingly supported by the principles of mechanobiology. It has been established that mechanical forces act as determinants of cellular identity through YAP/TAZ-mediated chromatin remodeling [16]. This signaling cascade serves as a molecular sensor of tissue strain, where activated YAP/TAZ interacts with epigenetic modifiers to maintain transcriptional fidelity [22].

Recent evidence reveals that aging is fundamentally characterized by the loss of organized chromatin interactions and the degradation of epigenetic information [23]. The author proposes that the neuro-mechanical feedback loop may serve to ‘reboot’ these chromatin interactions, thereby preserving the structural and functional vitality of the skin.

### 4.3. Evolutionary Neoteny and Systemic Resilience

Evolutionary neoteny as a bio-indicator of systemic resilience human neoteny—the retention of youthful features—is proposed as an evolutionary strategy linked to the Self-Domestication Hypothesis. Selection for reduced aggression is hypothesized to modulate the migration of neural crest cells (NCCs), resulting in reduced oxidative stress and distinct cranial morphology [20]. From this perspective, a youthful phenotype serves as a measurable indicator of systemic biological resilience and social fitness, rather than a mere aesthetic preference.

## 5. Systemic Molecular Interventions: Optimizing Biological Resilience

Recent breakthroughs in regenerative medicine have shifted the scientific paradigm toward the potential for systemic biological resetting [24]. A landmark study (2025) demonstrated that the systemic infusion of senescence-resistant, FOXO3-activated human mesenchymal progenitor cells into aged non-human primates (NHP) led to a significant mitigation of biological age markers across multiple organs, including potential cognitive rejuvenation [24]. This empirical success in primates provides a theoretical foundation for our inside–out strategy. Within this framework, visible health is proposed to be redefined as a measurable indicator of systemic physiological resilience—a manifestation of optimized systemic molecular crosstalk that integrates biological resilience, functional capacity, and biological indicators of well-being [25,26,27].

### 5.1. Metabolic Restoration Through NAD+ and Senolytics

Cellular energy homeostasis and clearance of inflammatory triggers are foundational to the proposed model of systemic modulation of aging markers. NAD+ levels have been observed to decline precipitously with age, potentially compromising mitochondrial function and sirtuin activity. Experimental evidence suggests that systemic supplementation with NAD+ precursors may restore SIRT1 activity, promoting keratinocyte differentiation in the skin and potentially protecting against UV-induced apoptosis by regulating p53 and PGC-1-α, potentially supporting the maintenance of the metabolic clock from within [2,3].

Complementary to this metabolic boosting, senolytic therapies are hypothesized to target the root of inflammaging by selectively inducing apoptosis in senescent cells through the inhibition of pro-survival pathways, such as BCL-2/BCL-XL [28,29]. By potentially reducing the systemic burden of the senescence-associated secretory phenotype (SASP), which is rich in pro-inflammatory cytokines like IL-1, IL-6, and MMPs, senolytics might mitigate the chronic degradation of dermal collagen and elastic fibers, thereby potentially “cooling” systemic inflammaging that accelerates visible skin aging [30].

### 5.2. GLP-1 Receptor Agonists and the Subcutaneous Paradox

Beyond their primary use in metabolic disorders, GLP-1 receptor agonists have emerged as candidates for potential systemic anti-aging agents [31]. In various models, these agonists activate AMPK signaling and suppress NF-kappaB-mediated oxidative stress, potentially mimicking the longevity benefits of caloric restriction. While they significantly reduce visceral adiposity—the primary source of systemic inflammaging—their use presents a unique clinical challenge known as the subcutaneous paradox. Global lipolysis induced by GLP-1 can contribute to the depletion of facial subcutaneous fat pads, which may paradoxically affect visible aging despite improved systemic metabolic health. This suggests the necessity of our integrated inside–out strategy, which would involve considering GLP-1 receptor agonists for systemic metabolic optimization while simultaneously employing local biostimulators, such as Poly-L-Lactic Acid (PLLA) [11], to potentially support the restoration of specific volume loss through YAP/TAZ-mediated mechanotransduction [16].

### 5.3. Levels of Evidence for Systemic Reversal

The current clinical applicability of these interventions is summarized in Table 1. While metabolic modulators like NAD+ and GLP-1 have reached the stage of human clinical observation for metabolic health, advanced regenerative therapies using stem cells and epigenetic modulators are currently demonstrating potential primarily in non-human primate models. Integrating these systemic modulators is hypothesized to ensure that local aesthetic procedures are performed on a resilient biological substrate, which might lead to more sustainable outcomes [6]. Integrating these systemic modulators with AI-based epigenetic clocks allows for more precise aging prediction and monitoring of intervention efficacy [32].

## 6. The Anatomy of Visible Aging: Integrated Phenotypes and Organ Crosstalk

In clinical aesthetic dermatology, visible aging has historically been reduced to changes in skin quality. However, from a systemic biological perspective, the appearance of the face and the body may be viewed as being produced through a multi-modal phenotype comprising distinct anatomical and biochemical components. Appearance thus potentially serves as a clinical readout of various internal “organ clocks.”

### 6.1. The Stratigraphy of Aging and Musculoskeletal Resorption

Visible aging manifests through distinct anatomical layers, each influenced by the exposome—including air quality, PM2.5, and UV radiation. Surface-level textures, such as fine lines, are primarily associated with impaired epidermal turnover and dermo-epidermal junction (DEJ) instability. In contrast, deep wrinkles and sagging result from the progressive collapse of dermal collagen, the atrophy of subcutaneous fat pads, and, significantly, the resorption of facial bone. Consequently, the aging face is hypothesized to reflect not just cutaneous decline but comprehensive aging of the entire musculoskeletal system, where the proposed “bone clock” may dictate the structural scaffolding of soft tissue.

### 6.2. Biochemical Hallmarks: Pigmentation and Dullness

Beyond structural changes, biochemical aging presents as alterations in color and translucency. Conditions such as melasma may represent a form of accelerated aging driven by DNA mutations, hormonal dysregulation, and dermal vascular dilation. Furthermore, dullness—a hallmark of biochemical aging characterized by yellowing— is hypothesized to be primarily driven by systemic oxidative stress, leading to protein carbonylation and the accumulation of advanced glycation end-products (AGEs) [34]. This systemic metabolic burden shifts the skin’s reflectance, potentially reflecting not only melanin accumulation but also a proposed “metabolic clock.”

### 6.3. Hair and Body Shape: Indicators of Kidney and Metabolic Clocks

External markers beyond the face provide further potential evidence of systemic organ health. Hair health, including volume and pigmentation, has been suggested to correlate with the proposed “kidney clock.” Specifically, preclinical observations suggest the decline in kidney-derived klotho is associated with premature aging phenotypes, including follicular atrophy and graying, positioning hair as a potential visible surrogate for renal longevity signaling [7,8,9]. Similarly, body shape—particularly the accumulation of visceral adiposity— may reflect the “metabolic clock.” The expansion of visceral fat is known to trigger the systemic release of pro-inflammatory adipokines, such as TNF-alpha and IL-6, which accelerate inflammaging and impair skin barrier integrity [4]. Thus, a comprehensive aesthetic assessment seeks to integrate these multi-modal signals to more accurately estimate the patient’s biological age.

## 7. Direct Dermal Interventions: Synergistic Local Signaling

While systemic optimization provides a biological foundation, aged skin is hypothesized to often require direct molecular “restarting.” Under our proposed framework, these local interventions are viewed not merely as physical treatments, but as potential mechanical and chemical epigenetic modulators that might convert exogenous signals into youthful gene expression patterns.

### 7.1. Chemical Signaling: Exosomes, Retinoids, and Metabolic Reprogramming

Direct epidermal and dermal renewal is primarily thought to be driven by paracrine and metabolic signaling. Stem-cell-derived exosomes serve as sophisticated vectors for regenerative information, delivering a cargo of bioactive proteins and microRNAs (e.g., miR-21, miR-29). In experimental models, these molecular cargoes have been shown to target Smad7 and PTEN within dermal fibroblasts, suppressing inflammaging and promoting high-fidelity collagen synthesis.

These paracrine signals are complemented by retinoids, which bind to nuclear RAR/RXR receptors. Upon translocation via CRABP2, these complexes are associated with normalized keratinocyte differentiation and inhibit MMP-1, 3, and 9, potentially mitigating mesenchymal drift. Furthermore, the clinical use of Poly-L-Lactic Acid (PLLA) has evolved into a proposed form of metabolic signaling; its degradation product, L-lactate, is hypothesized to act as a signaling molecule that binds to HCA2 receptors and promotes histone acetylation, potentially epigenetically reprogramming fibroblasts toward a non-inflammatory regenerative profile. Notably, klotho expression has been identified within hair follicle keratinocytes, where its decline may contribute to follicular aging and impaired regenerative capacity [9].

### 7.2. Mechanical Signaling: Hyaluronic Acid and Mechanotransduction

Hyaluronic acid (HA) fillers are hypothesized to not merely restore volume but physically stretch the extracellular matrix (ECM), stimulating fibroblast proliferation and matrix remodeling [35]. Based on mechanobiological models, this mechanical stress is thought to inhibit Lats1/2 kinase, triggering the nuclear translocation of the mechanotransducers YAP/TAZ, which may induce chromatin remodeling and allows the cell to potentially re-access youthful gene regulatory networks [16].

### 7.3. Energy-Based Epigenetic Modulation: RF, HIFU, and Photonic Technologies

High-intensity focused ultrasound (HIFU) and radiofrequency (RF) devices, traditionally categorized as thermal-inducing modalities, may act as sophisticated physical epigenetic modulators within our proposed framework. The immediate thermal contraction of collagen fibers induced by RF or HIFU generates sustained mechanical tension within the dermal matrix, which is hypothesized to be sensed by dermal fibroblasts via integrins, potentially triggering the nuclear translocation of key mechanotransducers, YAP/TAZ [16,36,37].

Recent evidence in various cellular models has established that YAP/TAZ coordinates the epigenetic reprogramming of transcriptional factors and their target enhancers [31,37]. As aging is characterized by the erosion of 3D chromatin architecture and a loss of chromatin accessibility [32], we propose that EBDs might counteract this structural decay. Once localized in the nucleus, YAP/TAZ has been observed in experimental settings to recruit SWI/SNF chromatin-remodeling complexes [33] to physically remodel the 3D chromatin landscape. This recruitment may facilitate the opening of condensed heterochromatin, enabling the transcriptional machinery to access gene-regulatory loci for COL1A1 and ELN. This mechanism suggests that energy-based stimuli may contribute to ‘rebooting’ cellular transcriptional fidelity by supporting the potential restoration of youthful 3D genomic organization.

Similarly, advanced photonic technologies, such as picosecond lasers utilizing laser-induced optical breakdown (LIOB), create precise intra-dermal vacuoles that are thought to initiate a rapid wound-healing cascade by upregulating HIF-1-α and VEGF [38]. These combined physical modalities suggest that EBDs may function not merely through “heating,” but by potentially modulating molecular switches to support the restoration of youthful epigenetic landscapes. While robust clinical validation in diverse human skin types is required to confirm these effects in human skin, this process may be optimized by post-procedure topical agents like dexpanthenol, which has been associated with accelerated barrier repair via Coenzyme A synthesis [39].

#### Critical Limitations and Translational Gaps

While the molecular framework presented here is supported by emerging evidence from non-human primate models and advanced in vitro studies, several critical limitations warrant consideration. First, the translational gap between controlled laboratory models and the highly variable human exposome remains significant; molecular responses observed in a vacuum may not fully reflect the complexities of human skin aging in real-world contexts. Second, although molecular biomarkers of “age reversal” are promising, long-term clinical safety and the durability of epigenetic remodeling induced by EBDs or systemic agents require further longitudinal human trials. Consequently, our proposed “inside-out” strategy is intended to serve as a theoretical foundation for future clinical validation rather than a definitive medical protocol.

### 7.4. Conclusion: Epigenetic Convergence of Local and Systemic Signals

**Figure 3:**
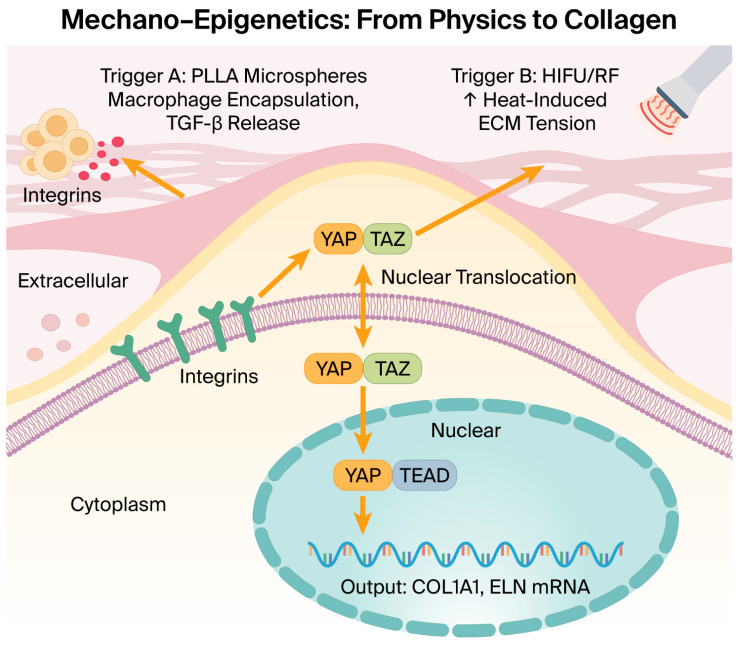
Proposed mechano-epigenetics pathway. Energy-based devices (HIFU/RF) and biostimulators (PLLA) are hypothesized to function as physical epigenetic modulators. Mechanical tension generated within the extracellular matrix is transduced via integrins and the cytoskeleton to the nuclear YAP/TAZ complex. This translocation is thought to recruit chromatin–remodeling factors, potentially increasing chromatin accessibility to upregulate COL1A1 and ELN (elastin) mRNA synthesis, thereby supporting the restoration of youthful transcriptional fidelity [16,33,37].

Ultimately, many dermal interventions are hypothesized to converge on a shared biological objective: supporting the potential for epigenetic rejuvenation. While systemic interventions (NAD+ [2], senolytics [26], and GLP-1 receptor agonists [28]) have been shown to optimize the internal milieu in various experimental models, these local therapies are hypothesized to provide the potential triggers needed to potentially “reboot” the skin’s cellular architecture. By integrating these modalities, the author proposes a transition toward a new paradigm of regenerative aesthetics, where aesthetic vitality is viewed as the result of a synchronized and epigenetically restored molecular landscape (Figure 3).

## 8. Conclusions: Regenerative Aesthetics and the Potential for Epigenetic Restoration

The future of aesthetic medicine lies in the seamless integration of systemic clock resetting and targeted regenerative signaling. The author is transitioning from an era of “topical camouflage” to one of integrative systemic skin health. Future interventions are hypothesized to combine oral metabolic modulators, such as NAD+ boosters and myokine mimetics, with local signaling therapies like PLLA, LIOB, and stem-cell-derived exosomes to achieve the potential mitigation of visible aging. Furthermore, the expanding understanding of how physical energies—including high-intensity focused ultrasound (HIFU), radiofrequency (RF), and coherent light—directly influence molecular pathways like YAP/TAZ signaling suggests that energy-based epigenetics will become a critical frontier in regenerative research.

This paradigm shifts our perspective of energy-based devices from simple thermal tools to sophisticated physical epigenetic modulators that might support the restoration of youthful transcriptional profiles. Ultimately, beauty may be viewed as not merely skin deep; it is the tangible expression of organ homeostasis and systemic biological resilience [40]. This perspective aligns with the concept of “health capital,” where health and appearance are viewed as assets that require continuous investment to maintain functional and social capacity [27]. This approach is further supported by sociological models that define appearance as a distinct biological indicator of personal well-being alongside economic, cultural, and social capital [41]. In this context, aesthetic vitality is emerging as a critical resource that is associated with social connection, self-efficacy, and economic opportunity. By treating the skin as a molecular readout of the systemic internal landscape, aesthetic medicine evolves into a discipline of comprehensive health management, where human flourishing is reflected in a rejuvenated and resilient phenotype [42].

## Figures and Tables

**Table 1:** Systemic interventions and levels of evidence for the mitigation of aging phenotypes.

Intervention Category	Specific Agents and Target Molecules	Level of Evidence	Observed/Expected Effects on Skin and Aging
NAD+ Boosters	NMN, NR (SIRT1, PARP1)	Human (RCT)	Improved epidermal barrier and metabolic resetting [2,3]
Senolytics	Dasatinib, Quercetin (BCL-2, p16)	Human (Phase I/II)	Reduction in SASP and clearance of “zombie” cells [28,29]
GLP-1 RA	Semaglutide (GLP-1R, AMPK, NF-κB)	Human (Clinical)	Reduction in systemic inflammaging [31]
Stem Cells/Exosomes	MSC-derived EVs (miR-21, miR-29)	Primate (2025)	Systemic modulation and mitigation of epigenetic aging markers [24]
Biostimulators (PLLA/HA)	PLLA, HA (YAP/TAZ, Lats1/2, HCA2)	Primate/Pre-Clinical	Mechanotransduction and potential metabolic modulation [11,33]

Note: NMN, nicotinamide mononucleotide; NR, nicotinamide riboside; BCL-2, B-cell lymphoma 2; SASP, senescence-associated secretory phenotype; PLLA, poly-L-lactic acid; GLP-1 RA, glucagon-like peptide-1 receptor agonist; SCFA, short-chain fatty acid; HIFU, high-intensity focused ultrasound; RF, radiofrequency; YAP/TAZ, Yes-associated protein/transcriptional coactivator with PDZ-binding motif; SWI/SNF, SWItch/Sucrose Non-Fermentable.

## Data Availability

Not applicable.

## Abbreviations

The following abbreviations are used in this manuscript:

Abbreviation	Full Term
SCFA	Short-Chain Fatty Acids
LPS	Lipopolysaccharides
ECM	Extracellular Matrix
SASP	Senescence-Associated Secretory Phenotype
PLLA	Poly-L-Lactic Acid
HA	Hyaluronic Acid
NHP	Non-Human Primate
GLP-1	Glucagon-Like Peptide-1
EBD	Energy-Based Device
HIFU	High-Intensity Focused Ultrasound
RF	Radiofrequency
LIOB	Laser-Induced Optical Breakdown
YAP/TAZ	Yes-Associated Protein/Transcriptional Coactivator with PDZ-binding Motif
